# Microsite Differentiation Drives the Abundance of Soil Ammonia Oxidizing Bacteria along Aridity Gradients

**DOI:** 10.3389/fmicb.2016.00505

**Published:** 2016-04-18

**Authors:** Manuel Delgado-Baquerizo, Fernando T. Maestre, David J. Eldridge, Brajesh K. Singh

**Affiliations:** ^1^Hawkesbury Institute for the Environment, Western Sydney University, PenrithNSW, Australia; ^2^Área de Biodiversidad y Conservación, Departamento de Biología y Geología, Física y Química Inorgánica, Escuela Superior de Ciencias Experimentales y Tecnología, Universidad Rey Juan CarlosMóstoles, Spain; ^3^School of Biological, Earth and Environmental Sciences, University of New South Wales, SydneyNSW, Australia; ^4^Global Centre for Land-Based Innovation, Western Sydney University, PenrithNSW, Australia

**Keywords:** nitrogen cycle, drylands, biocrusts, nitrifiers, nitrification

## Abstract

Soil ammonia oxidizing bacteria (AOB) and archaea (AOA) are responsible for nitrification in terrestrial ecosystems, and play important roles in ecosystem functioning by modulating the rates of N losses to ground water and the atmosphere. Vascular plants have been shown to modulate the abundance of AOA and AOB in drylands, the largest biome on Earth. Like plants, biotic and abiotic features such as insect nests and biological soil crusts (biocrusts) have unique biogeochemical attributes (e.g., nutrient availability) that may modify the local abundance of AOA and AOB. However, little is known about how these biotic and abiotic features and their interactions modulate the abundance of AOA and AOB in drylands. Here, we evaluate the abundance of *amoA* genes from AOB and AOA within six microsites commonly found in drylands (open areas, biocrusts, ant nests, grasses, nitrogen-fixing shrubs, and trees) at 21 sites from eastern Australia, including arid and mesic ecosystems that are threatened by predicted increases in aridity. Our results from structural equation modeling suggest that soil microsite differentiation alters the abundance of AOB (but not AOA) in both arid and mesic ecosystems. While the abundance of AOA sharply increased with increasing aridity in all microsites, the response of AOB abundance was microsite-dependent, with increases (nitrogen-fixing shrubs, ant nests), decreases (open areas) or no changes (grasses, biocrusts, trees) in abundance with increasing aridity. Microsites supporting the highest abundance of AOB were trees, nitrogen-fixing shrubs, and ant nests. These results are linked to particular soil characteristics (e.g., total carbon and ammonium) under these microsites. Our findings advance our understanding of key drivers of functionally important microbial communities and N availability in highly heterogeneous ecosystems such as drylands, which may be obscured when different soil microsites are not explicitly considered.

## Introduction

Arid, semi-arid, and dry-sub humid ecosystems (drylands) constitute the planet’s largest biome, and support over 38% of the global human population (Reynolds et al., 2007; Schimel, 2010). A major feature of dryland ecosystems is their spatial heterogeneity (Tongway et al., 2001; Maestre and Cortina, 2002). Drylands are characterized by a sparse coverage of plants, which are separated by open areas devoid of perennial vegetation. Plant patches include a wide variety of vegetation types such as grasses, nitrogen (N)-fixing shrubs, and trees, while open areas are often covered by biocrusts (soil communities dominated by mosses, lichens, and cyanobacteria) and support the nests and burrows of soil arthropods (Belnap et al., 2001; Bonachela et al., 2015). Recent studies suggest that each of these soil surface attributes has unique effects on microbial communities and ecosystem processes in drylands (e.g., mineralization; Castillo-Monroy et al., 2010; Hortal et al., 2013; Bonachela et al., 2015; Delgado-Baquerizo et al., 2015). However, until now, their effects have largely been evaluated separately. To date, no previous research has simultaneously evaluated how multiple soil surface features affect both microbial communities and soil variables in drylands, nor have any studies explored the likely mechanisms behind the observed microsite-specific effects on these ecosystem attributes.

Dryland ecosystems are highly vulnerable to climate change and desertification processes (Reynolds et al., 2007; Maestre et al., 2012). Recent studies suggest that the increase in aridity for the late 21st century forecasted for most drylands (Dai, 2013; Feng and Fu, 2013; Huang et al., 2016) will have negative impacts on the cover and richness of vascular vegetation (Maestre et al., 2012; Vicente-Serrano et al., 2012). Conversely, such declines in plant cover could expand the area occupied by open areas and biocrusts by increasing the surface available for colonization and growth of their constituent organisms (Belnap et al., 2001; Thomas et al., 2011). In addition to climate change, human activities such as overgrazing are also expected to have substantial negative effects on plants and biocrusts in drylands (Fuhlendorf and Engle, 2001; Eldridge et al., 2013). These global change drivers will shift the relative abundance of different microsites in these areas (Whitford, 2002; Escolar et al., 2012; Vicente-Serrano et al., 2012), likely altering their microsite-specific effects on microbial communities and ecosystem functioning. Thus, understanding how different soil microsites affect particular microbial communities and associated ecosystem functions is of paramount importance if we are to predict how dryland ecosystems will respond to global change.

Nitrogen is one of the most important factors limiting net primary productivity and organic matter decomposition in drylands (Schlesinger and Bernhardt, 2013). The availability of N for plants and microbes is regulated mainly by particular microbial guilds (see Robertson and Groffman, 2007 for a review). For example, the critical process of autotrophic nitrification, which converts ammonium to nitrite, is driven principally by the abundance of ammonia-oxidizing bacteria (AOB) and archaea (AOA; Nicol et al., 2008; Verhamme et al., 2011). Understanding the mechanisms that control the abundance of these microorganisms and their effects on soil N availability is thus critical for understanding and managing soil fertility and ecosystem productivity (Robertson and Groffman, 2007). The abundance of AOA and AOB in soils is known to be differentially affected by factors such as pH, organic matter quality and substrate availability (He et al., 2007; Nicol et al., 2008; Wessén et al., 2010; Rasche et al., 2011; Verhamme et al., 2011). Different dryland microsites (vascular plants, open areas, biocrusts, and insect nests), which have unique effects on soil attributes such as nutrient availability, organic matter quality and content (Castillo-Monroy et al., 2010; Hortal et al., 2013; Bonachela et al., 2015; Delgado-Baquerizo et al., 2015), could provide potentially different niches for AOA and AOB. Recent studies have shown that vascular plants can modulate the abundance of soil AOA and AOB along aridity gradients (Delgado-Baquerizo et al., 2013b), and that variations in the abundance of AOA and AOB drive variations in nitrification rates (Adair and Schwartz, 2008; Delgado-Baquerizo et al., 2013b; Hortal et al., 2013; Marusenko et al., 2013) in drylands. However, little is known about the role of plants and other biotic and abiotic features, such as insect nests and biocrusts, as modulators of the abundance of AOA and AOB in response to increasing aridity.

Here, we explore the effects of six markedly different microsites (open areas, biocrusts, ant nests, grasses, shrubs, and trees) on the abundance of *amoA* genes from AOB and AOA and on nitrate availability, and evaluate the mechanisms underlying microsite-specific effects on these variables at 21 sites along an aridity gradient in eastern Australia. As AOB have been previously reported to prefer high substrate concentration under plant canopies (Martens-Habbena et al., 2009; Verhamme et al., 2011; Delgado-Baquerizo et al., 2013b), we hypothesized that their abundance will peak in microsites with the highest ammonium availability. Conversely, because AOA abundance has been previously reported to increase with aridity in both open areas and under vascular plant canopies (Delgado-Baquerizo et al., 2013b), we predicted that AOA abundance would be microsite-independent and be driven by particular changes in soil properties derived from increasing aridity.

## Materials and Methods

### Study Area

**FIGURE 1:**
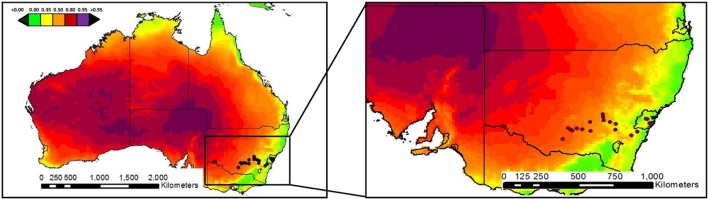
**Locations of the study sites.** Color patterns indicate aridity (1 – aridity index) gradients. Aridity increases from green to purple in the graphs.

This study was carried out in 21 sites from eastern Australia (**Figure 1**; **Supplementary Table S1**). Locations for this study were chosen to represent a wide range of aridity conditions, including arid (arid and semiarid; *n* = 12; aridity index < 0.50) and mesic (dry-subhumid and humid; *n* = 9; aridity index > 0.50) ecosystems. Total annual precipitation and mean temperature ranged from 280 to 1167 mm and from 12.8 to 19.5°C, respectively. The sites surveyed encompass a wide variety of vegetation types, including grasslands, shrublands, savannas, dry seasonal forests, and open woodlands dominated by trees. Perennial vegetation cover ranged between 18 and 98%, and was dominated by *Eucalyptus* spp., *Acacia* spp., and *Austrodanthonia* spp. All our sites had a well-developed biocrust community dominated by mosses (*Desmatodon convolutus*, *Barbula calycina*, *Didymodon torquatus*, and *Gemmabryum* spp.).

### Sampling Design and Measurements

Soil sampling was carried out in March 2014 according to a standardized sampling protocol. A 30 m × 30 m plot representative of the dominant vegetation was established. The cover of the most abundant microsites (open areas, biocrust, ant nest entrances, grasses, N-fixing shrubs, and trees) at each site was measured using four 30-m transects and the line-intercept method, as described in Maestre et al. (2012). Aridity was determined as 1-aridity index [AI], where AI = precipitation/potential evapotranspiration (UNEP, 2012). Data of the aridity index were obtained from the global aridity map of the FAO (2014).

At each site, three soil cores (0–5 cm depth) were collected under the six most common microsites: open areas, biocrusts, ant nests, grasses (*Austrodanthonia* spp.), N-fixing shrubs (*Acacia* spp.), and trees (*Eucalyptus* spp.). Soil cores were then mixed to get a composite soil sample per microsite (six samples) in each of the sites. A minimum separation distance of 1 m between samples, and between these and plant patches was maintained to remove potential sources of non-independence between samples (Delgado-Baquerizo et al., 2013a). Following field sampling, the soil was sieved (2 mm mesh) and separated into two fractions. A fraction was immediately frozen at -20°C for quantifying the abundance of *amoA* genes from AOB and AOA (hereafter, AOA and AOB abundance). The other fraction was air-dried and stored for 1 month before physicochemical analyses.

We measured organic C, total N, pH, ammonia, and nitrate availability in all the soil samples. We selected these soil properties and nutrient variables because they are largely known to be important drivers of the abundance of AOA and AOB in terrestrial ecosystems (He et al., 2007; Nicol et al., 2008; Verhamme et al., 2011). The concentration of soil organic C was determined as described in Anderson and Ingram (1993). Soil total N was measured with a CN analyzer (Leco CHN628 Series, LECO Corporation, St Joseph, MI, USA). Soil pH was measured for all of the soil samples with a pH meter in soil and water suspension. Ammonium and nitrate were colorimetrically analyzed (Sims et al., 1995) from K_2_SO_4_ 0.5 M soil extracts using a 1:5 soil: extract ratio as described in Jones and Willett (2006). The main soil properties for the different microsites and aridity conditions used in this study are shown in **Supplementary Table S1**.

### Molecular Analyses

We measured the abundance of ammonia oxidizing bacteria (AOB) and archaea (AOA) at each microsite and location using quantitative PCR (qPCR). Soil DNA was extracted from 0.5 g of defrosted soil samples using the Powersoil DNA Isolation Kit (Mo Bio Laboratories, Carlsbad, CA, USA) according to the instructions provided by the manufacturer. qPCR reactions were conducted in triplicate using 96-well plates on an CFX96 Touch^TM^ Real-Time PCR Detection System (Foster city, CA, USA). The *amoA* genes of AOB and AOA were amplified using the primers *amoA1F* (GGGGTTTCTACTGGTGGT)/*amoA2R* (CCCCTCKGSAAAGCCTTCTTC) and *Arch-amoAF* (STAATGGTCTGGCTTAGACG)/*Arch amoAR* (GCGGCCATCCATCTGTATGT), respectively, as described previously by Rotthauwe et al. (1997) and Francis et al. (2005). Efficiencies for all quantification reactions were higher than 90%, with *R*^2^ values ranging from 0.90 to 0.99. Standards were run in triplicate in each assay, and our standard calibration curve was developed using a serial 10^-3^ and 10^-9^ dilution from 30 ng μl^-1^. Samples fell within the limits of our standard curve, hence within the detection limit. We generated melting curves for each run to verify product specificity by increasing the temperature from 55 to 95°C. Melting curve analyses resulted in a single peak, confirming the specificity of all amplicons. Actual values of AOA and AOB abundances for the different microsites and aridity conditions used in this study are available in **Supplementary Table S1**.

### qPCR Standard Curve Preparation

The AOA and AOB primers described above were used to amplify *amoA* genes from DNA extracted from composite soil samples. In parallel, both PCR products were cloned into *Escherichia coli* using a TOPO TA cloning kit (Invitrogen) according to the manufacturer’s instructions. One specific clone was selected for AOA and AOB cultures in order to generate the standard curves. Plasmid DNA was extracted with a Plasmid Mini Kit (Invitrogen), and the insert was sequenced using M13F and M13R primers to check that AOA and AOB were correctly inserted into their respective plasmids (sequences from selected AOA and AOB clones are available in **Supplementary Table S1**). These sequences were compared to known *amoA* genes in the GenBank database (http://www.ncbi.nlm.nih.gov) using BLAST. This analysis showed that the sequences were >99% similar to known AOA and AOB genes.

### Statistical Analyses

We tested for differences across aridity conditions (arid vs. mesic) and microsites (open areas, biocrusts, ant nests, grasses, N-fixing shrubs, and trees) for the abundance of *amoA* genes from AOB and AOA with a two-way ANOVA, with microsite and aridity conditions as fixed factors. Prior to analyses, abundance of *amoA* genes from AOB and AOA and nitrate were log-transformed to improve normality. We conducted *post hoc* analyses (Tukey test) to explore differences in AOB, AOA and nitrate among microsites for those analyses where microsite effect was significant. When interactions between aridity conditions and microsites were significant, we carried our separate *post hoc* analyses for mesic and arid ecosystems. Spearman correlations were used to evaluate the relationship between aridity and both the relative abundance of the different microsites and the abundance of *amoA* genes from AOB and AOA in each microsite. All these analyses were carried out using SPSS for Windows, version 15.0 (SPSS Inc., Chicago, IL, USA).

We used structural equation modeling (SEM; Grace, 2006) to evaluate direct and indirect relationships between the different microsites (open areas, biocrusts, ant nests, grasses, N-fixing shrubs, and trees), aridity (1-aridity index), soil properties (pH, C:N ratio, and soil C) and substrate (ammonium) on the abundance of *amoA* genes from AOB and AOA and nitrate availability. Unlike regression or ANOVA, SEM offers the ability to separate multiple pathways of influence and view them as a system, and thus is useful for investigating the complex networks of relationships found in ecosystems (Grace, 2006; Eisenhauer et al., 2015). Thus, this approach was the appropriate tool to evaluate our hypotheses because it allowed us to assess whether microsite effects on AOA and AOB abundance were directly or indirectly driven via nutrient availability and soil properties. The first step in SEM requires establishing an *a priori* model based on the known effects and relationships among the drivers of AOB and AOA abundance and nitrification process. Some data manipulation was required prior to modeling. We examined the distributions of all of our endogenous variables, and tested their normality. Soil C, C:N ratio, pH, ammonium, AOA and AOB abundances were log-transformed to improve normality. In these models, the different microsites (open areas, biocrusts, ant nests, grasses, N-fixing shrubs, and trees) are categorical exogenous variables (Grace, 2006) with two levels: 0 (specific microsite) and 1 (remaining microsites). This approach allowed us to compare the effect of a particular microsite (e.g., ant nests) on the abundance of AOA and AOB compared with the average of the remaining microsites. Categorical exogenous variables can be used in SEM because distributional assumptions do not apply to them (Grace, 2006). The combined effects of nitrifier (AOA and AOB) abundances on nitrate availability were included in our model as a composite variable (Grace, 2006). With a good model fit (see below), we could confidently interpret the path coefficients of the model and their associated *P*-values. A path coefficient is analogous to the partial correlation coefficient, and describes the strength and direction of the relationship between two variables (Grace, 2006). We parameterized our models after data manipulation and used our datasets to test them for their overall goodness of fit. There is no single universally accepted test of overall goodness of fit for SEM that is applicable in all situations regardless of sample size or data distribution. Here, we used the chi-squared test (χ^2^; the model has a good fit when 0 ≤ χ^2^ ≤ 2 and 0.05 < *P* ≤ 1.00) and the root mean square error of approximation (RMSEA; the model has a good fit when *RMSEA* 0 ≤*RMSEA*≤ 0.05 and 0.10 < *P* ≤ 1.00). Additionally, and because some variables were not normal, we confirmed the fit of the model using the Bollen-Stine bootstrap test (the model has a good fit when 0.10 < bootstrap *P* ≤ 1.00; Schermelleh-Engel et al., 2003).

We calculated the standardized total effects of microsite, aridity, soil properties and substrate on abundance of AOA and AOB and nitrate availability. The net influence that one variable has upon another is calculated by summing all direct and indirect pathways between the two variables. If the model fits the data well, the total effect should approximately be equivalent to the bivariate correlation coefficient for that pair of variables (Grace, 2006). All the SEM analyses were conducted using AMOS 20.0 (IBM SPSS, Chicago, IL, USA).

## Results

**FIGURE 2:**
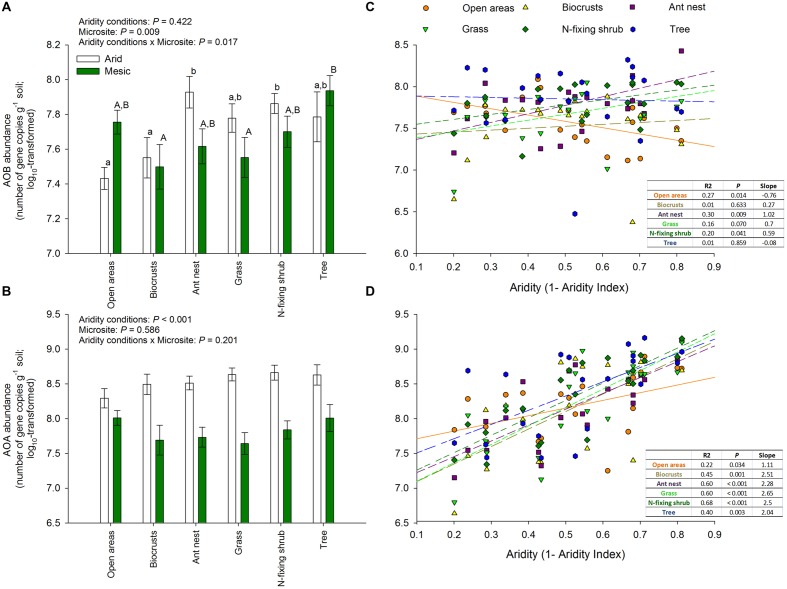
**Changes in the abundance of *amoA* genes from AOB and AOA across different levels of aridity.(A,B)** Show mean ± SE, *n* = 12 and 9 for arid and mesic ecosystems, respectively. Upper case letters indicate differences between microsites (Tukey *post hoc* test after ANOVA). When interactions between aridity conditions and microsite were found, we conducted *post hoc* analyses independently for arid and mesic ecosystems. In this case, lower and upper case letters are used to show microsite differences separately for arid and mesic ecosystems. **(C,D)** Represent linear regressions between aridity and of the abundance of *amoA* genes from AOB and AOA across different microsites. The solid and dashed lines in **(C,D)** represent the fitted linear regressions.

**FIGURE 3:**
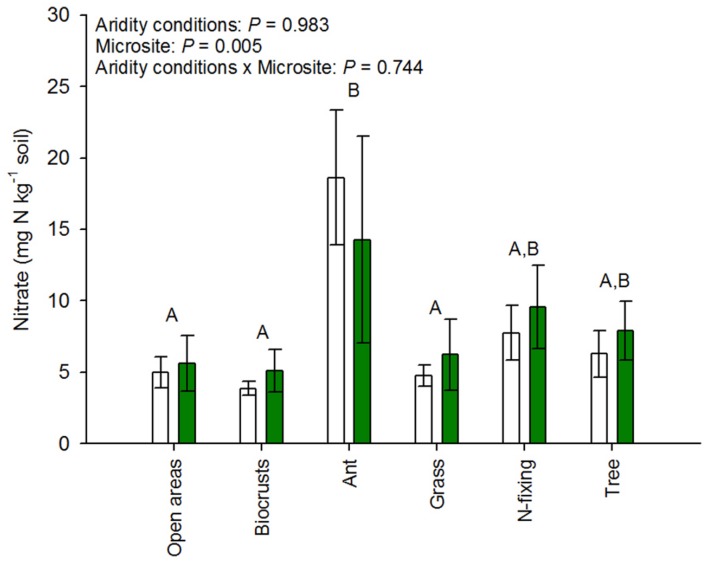
**Nitrate availability in the different microsites studied.** Data are mean ± SE, *n* = 12 and 9 for arid and mesic ecosystems, respectively. Upper case letters indicate differences between microsites (Tukey *post hoc* test after ANOVA).

The cover of open areas, biocrusts, ant nests, grasses, N-fixing shrubs, and trees ranged from 0–43%, 0–73%, 0–1%, 0–25%, 0–10%, and 0–70%, respectively. The cover of biocrusts increased (ρ = 0.67, *P* = 0.001), while that of ant nests and trees decreased along the aridity gradient studied (ρ_ant nests_ = -0.55, *P* = 0.010; ρ_trees_ = -0.74, *P* < 0.001). The cover of the remaining microsites did not vary significantly with aridity, though clear trends were observed in some cases (ρ_bare ground areas_ = 0.35, *P* = 0.120; ρ_grasses_ = -0.41, *P* = 0.064; ρ_N-fixing shrubs_ = -0.40, *P* = 0.069). Our results revealed important differences in the abundance of *amoA* genes from AOB (but not AOA) among microsites in both arid and mesic ecosystems (**Figure 2**). Ant nests, N-fixing shrubs and trees showed the highest AOB abundance (**Figure 2A**). This was particularly evident for ant nests and N-fixing shrubs under the most arid conditions, as indicated by the significant aridity conditions × microsite interaction (*P* < 0.001; **Figure 2A**). Thus, this interaction provides evidence that the size effect of microsite on AOB differ between xeric and mesic ecosystems. Biocrusts and open areas had the lowest AOB abundance (**Figure 2A**), particularly in the arid sites (aridity conditions × microsite interaction: *P* < 0.001; **Figure 2A**). We did not find significant differences between microsites for AOA abundance, which consistently showed the highest abundance in the most arid parts of the gradient (*P* < 0.001; **Figure 2B**). Interestingly, the abundance of *amoA* genes from AOA increased with increasing aridity in all microsites (**Figure 2D**), but the response of AOB to aridity was microsite dependent, with increases (grasses, N-fixing shrubs and ant nests), decreases (open areas) or no changes (biocrusts and trees) in their abundance with increasing aridity (**Figure 2C**). We found the highest nitrate availability under ant nests, followed by trees and N-fixing shrubs (*P* < 0.001; **Figure 3**). Differences between arid and mesic ecosystems were not observed for this variable (*P*> 0.05; **Figure 3**).

**FIGURE 4:**
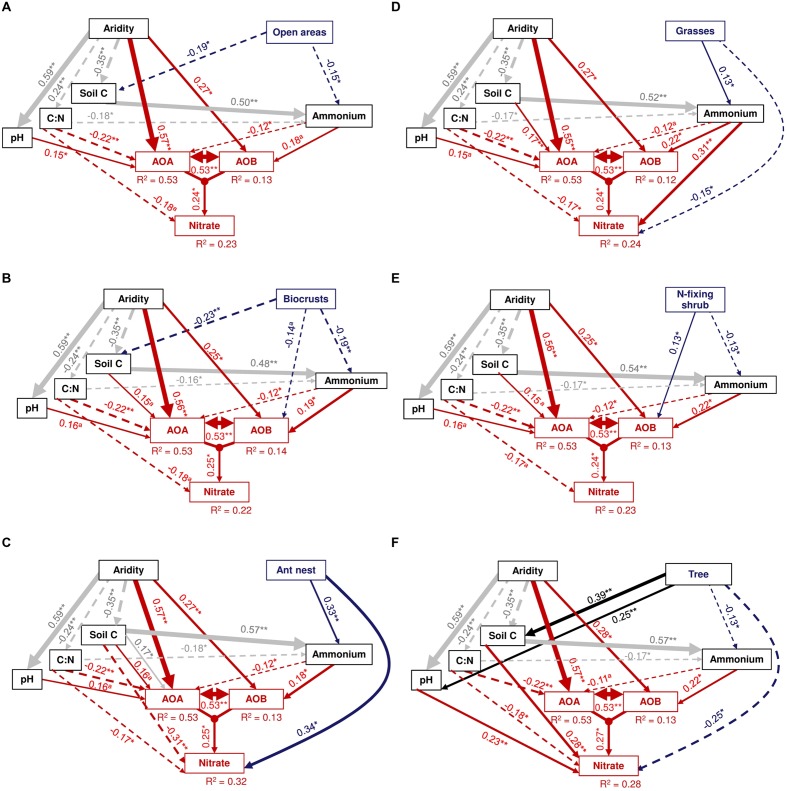
**Results from structural equation modeling showing the direct and indirect effects of aridity and different microsites on the abundance of AOB and AOA and on nitrate availability.** Each panel represents the model used for each microsite (indicated in the upper right box of each model). Numbers adjacent to arrows are standardized path coefficients, analogous to relative regression weights, and indicative of the effect size of the relationship. Continuous and dashed arrows indicate positive and negative relationships, respectively. The width of arrows is proportional to the strength of path coefficients. Double-headed arrows between AOA and AOB indicate that the abundance of AOA can influence that one from AOB and *viseversa*. The proportion of variance explained (*R*^2^) appears above every response variable in the model. Significance levels are as follows: ^∗^*P* < 0.05, ^∗∗^*P* < 0.01, and ^∗∗∗^*P* < 0.001.

Our *a priori* SEM model was satisfactorily fitted to our data, as suggested by non-significant χ^2^ values (χ^2^ = 0.6–4.2; *P* = 0.37–0.98; *df* = 4), non-parametric Bootstrap *P-*values ranging from 0.46 to 0.97 and by values of RMSEA ranging from 0.00 to 0.03 (0.53 < *P* < 0.98; **Figure 4**). Most microsite effects (i.e., open areas, ant nests, grasses, and trees) on the abundance of *amoA* genes from AOB were indirectly driven by variations in soil C and ammonium availability, suggesting that these predictors adequately explained the abundance of AOB beneath these microsites. Contrary to this, we found a predominant direct positive and negative effect of N-fixing shrubs (**Figure 4E**) and biocrusts (**Figure 4B**) on the abundance of AOB, respectively, indicating that other unmeasured factors may have driven the indirect effects of these microsites on the abundance of *amoA* genes from AOB. Open areas and biocrusts showed a negative direct effect on soil C and ammonium compared with the other microsites, promoting an indirect negative effect on the abundance of AOB and on nitrate availability (via soil C and ammonium; **Figures 4A,B**). Conversely, ant nest and grasses had an indirect positive effect on AOB abundance via their influence on that of ammonium (**Figures 4C,D**), while trees had an indirect positive effect on AOB abundance by affecting soil C, hence the availability of ammonium in soil (**Figure 4F**). For AOA, our SEM results revealed that aridity had the highest direct positive effect on the abundance of *amoA* genes from AOA (**Figure 4**); aridity also had an indirect positive effect on these microorganisms by reducing both the C:N ratio (negatively related to the abundance of AOA), and the amount of soil C (positively related to ammonium; **Figure 4**) and by enhancing soil pH (positively related to AOA abundance; **Figure 4**).

**FIGURE 5:**
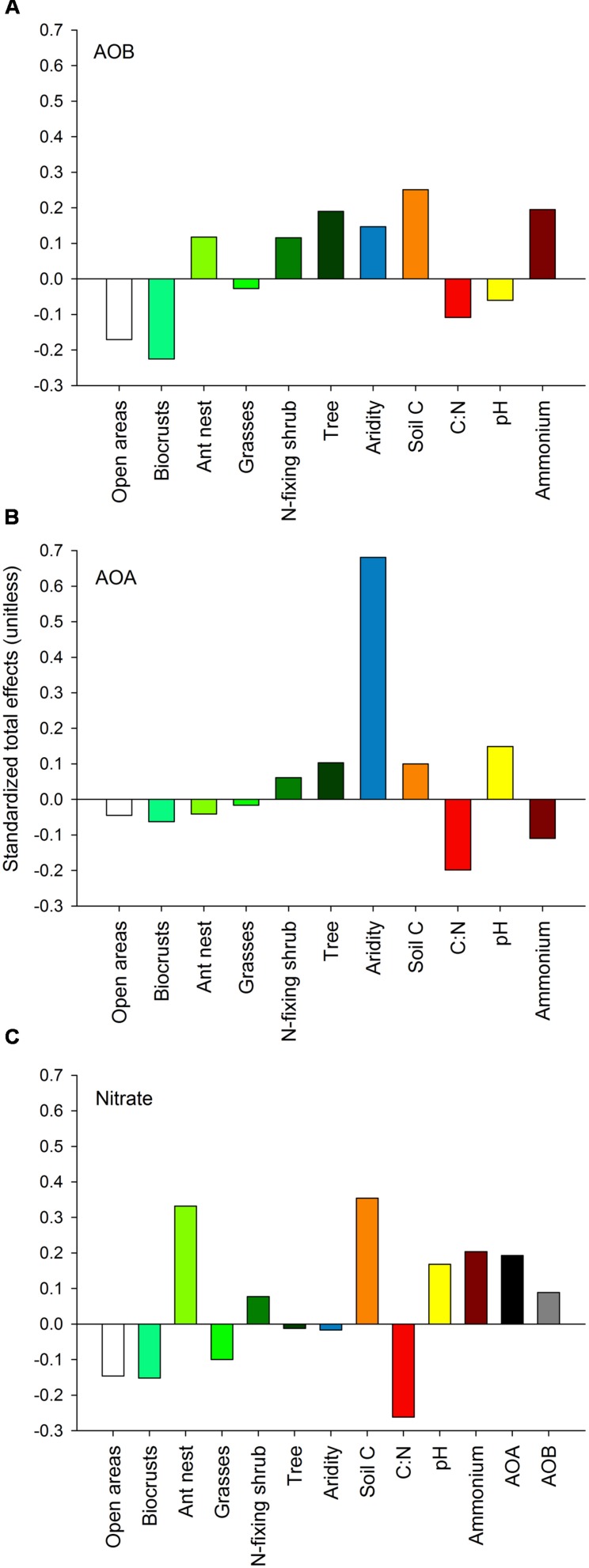
**Standardized total effects (direct plus indirect effects) derived from the structural equation modeling, including the effects of aridity and the different microsites evaluated on the abundance of AOB **(A)** and AOA **(B)** and on nitrate availability **(C)**.** The total effects of aridity, soil C, pH, C:N and ammonium on AOB and AOA abundance and nitrate availability were averaged from all six models in **Figure 4**.

Our SEM models supported the microsite dependence of AOB (but not AOA) abundance in the ecosystems studied, and indicated that both aridity and microsite differentiation were the most important factors predicting the abundance of AOA and AOB, respectively (**Figure 5**). Ant nests, N-fixing shrubs and trees had a total positive effect (sum of direct and indirect effects from SEM) on the abundance of AOB, while open areas and biocrusts showed the highest negative total effect compared to other microsites (**Figure 5A**). Nitrogen-fixing shrubs and trees were the only microsites showing positive total effects on the abundance of AOA, but the magnitude of these effects was about seven-times lower than the total positive effect from aridity on such abundance (**Figure 5B**).

The abundance of both *amoA* genes from AOA and AOB had positive total effects on nitrate availability (**Figure 5C**). Ant nests (+), soil C (+), C:N ratio (-), ammonium (+) and both AOA (+) and AOB (+) abundance were the main predictors of nitrate availability (**Figures 4** and **5**). Aridity had a negative, but weak (i.e., close to 0), effect on nitrate concentration, while the positive effect of AOA abundance on nitrate was higher than that of AOB abundance (**Figure 5**).

## Discussion

### Microsite-Specific Effects on the Abundance of AOB and AOA

Our study provides empirical evidence that microsite differen-tiation modulates the abundance of particular groups of microorganisms, such as AOB, in response to increases in aridity. This pattern was not found for AOA, as their abundance was driven mainly by aridity, regardless of the microsite considered. Strikingly, ant nests were one of the microsites with the highest abundance of AOB. Ants are one of the most widespread insect groups globally (Pêtal, 1998; Lenoir et al., 2001), and their activity during nest construction has major effects on multiple soil properties, including clay mineralogy, water infiltration and retention and nutrient cycling (Lenoir et al., 2001; Bonachela et al., 2015; Wu et al., 2015). Ant nests have been reported to support higher concentrations of inorganic N (ammonium and nitrate) and phosphorus due to the accumulation of animal and plant litter (Lenoir et al., 2001; Bonachela et al., 2015; Wu et al., 2015), which can be used by the plants growing near them (Sternberg et al., 2007; Wagner and Nicklen, 2010). They have also been observed to promote mineralization and nitrification processes in soils (Pêtal, 1998; Lenoir et al., 2001). This is particularly important in drylands, where N is one of the most important factors limiting the growth of plants and microorganisms (Austin et al., 2004; Schlesinger and Bernhardt, 2013). Thus, ant nests may act as “resource islands” (*sensu* Reynolds et al., 1999), providing suitable habitat for particular groups of microbes such as AOB, which support the high nitrification rates and nitrate availability reported here and in other studies (Pêtal, 1998; Lenoir et al., 2001; Wagner and Jones, 2006).

We found that the effects of vascular plants on the abundance of AOB varied depending on their functional attributes. The observed differences in the abundance of AOB between microsites may have been due to their different effects on microclimate and soil properties. The surface of soils close to and under trees and N-fixing shrubs receives greater levels of litter than soils adjacent to bare ground, biocrusts or small grasses (Vesterdal et al., 2012; Travers and Eldridge, 2015), and has lower temperature and higher infiltration rates that may lead to improved soil moisture conditions (Breshears et al., 1997; Berdugo et al., 2014; Eldridge et al., 2015). Overall, these environmental changes may provide a refuge for AOB in drylands. Interestingly, although small grasses had a lower effect on AOB abundance than other vascular plants, they still provide a better refuge for AOB than open areas and biocrusts, a response likely linked to the higher ammonium content found under grasses. Our results build up those from Delgado-Baquerizo et al. (2013b), who found that vascular plants can modulate the abundance of AOB in drylands, by emphasizing the role of different plant functional types on the abundance of these microorganisms.

Contrary to the results for AOB abundance, which was highly microsite-dependent, abundance of AOA increased along the aridity gradient studied, and was not affected by any of the microsites considered. Our results mimic those from Delgado-Baquerizo et al. (2013b), who also found increases in the abundance of AOA along an aridity gradient in Mediterranean grasslands dominated by *Stipa tenacissima*. Other studies have also reported high abundance of AOA in drylands (Adair and Schwartz, 2008; Marusenko et al., 2013). Remarkably, our results further suggest that this effect is microsite-independent, and that aridity can be the best predictor for AOA abundance in the studied ecosystems. These results further support the notion that AOA often occupy those niches with more extreme conditions (i.e., low water and nutrient availability), where they usually outcompete AOB (Valentine, 2007; Adair and Schwartz, 2008; Moin et al., 2009; You et al., 2009).

### Microsite as a Modulator of the Abundance of AOB and AOA in Response to Aridity

Our results indicate that microsite differentiation not only modified the abundance of AOB, but also their responses to aridity. While the abundance of AOA increased with increasing aridity, irrespective of the particular microsite, the response of AOB abundance was microsite dependent, with increases (grasses, N-fixing shrubs, and ant nests), decreases (open areas) or no changes (biocrusts and trees) in abundance with increasing aridity (**Figures 2C,D**). Predicted increases in aridity for the late 21st century in most drylands (Dai, 2013; Feng and Fu, 2013) will negatively impact upon vascular vegetation cover in drylands (Maestre et al., 2012; Vicente-Serrano et al., 2012), and this may increase the proportion of suitable habitat for biocrusts, and thus their cover (Belnap et al., 2001; Thomas et al., 2011). Accordingly, we found a significant decrease in the cover of trees (and clear trends in the same direction in the cover of N-fixing shrubs and grasses), and an increase in the cover of biocrusts, with aridity. The decrease in plant cover and the increase of open areas will likely increase the abundance of AOA at the expenses of AOB due to the high resistance to water and nutrient stresses of the former (Adair and Schwartz, 2008; Verhamme et al., 2011).

Overall, all of our models indicate that the abundance of AOA and AOB is positively related to nitrate availability in our study sites, suggesting that autotrophic ammonia oxidation may be extremely important for N cycling in dryland ecosystems. This is particularly true for AOA abundance, which showed the highest total positive effect on nitrate availability in our models (**Figure 3**). Thus, considering the positive effect of aridity on AOA abundance and the importance of the positive effect of AOA on nitrate availability, our results suggest that AOA abundance could contribute to buffer the direct negative effects of aridity increasing on nitrate availability in drylands (**Figure 5C**; Delgado-Baquerizo et al., 2016). The role of these microorganisms in nitrification is largely supported in laboratory studies (e.g., Verhamme et al., 2011), but our study confirms the importance of the relationship between ammonia-oxidizing microbe abundance and the availability of nitrate under field-based conditions in drylands. Indeed, these results are consistent with reports showing that the relative dominance of nitrate will increase with increasing aridity (Schlesinger et al., 1990; Delgado-Baquerizo and Gallardo, 2011), and suggest that any increase in aridity resulting from climate change (Dai, 2013; Feng and Fu, 2013) may promote losses in nitrate to both the atmosphere and underground water (Robertson and Groffman, 2007; Delgado-Baquerizo et al., 2016).

### Mechanisms that Account for Microsite-Specific and Aridity Effects on the Abundance of AOB and AOA

Our SEM approach allowed us to identify the most likely mechanisms controlling microsite and aridity effects on the abundance of AOB and AOA. For example, microsite differentiation indirectly alters the abundance of AOB via organic C and ammonium availability. Thus, the positive effects of ant nests and grasses on the abundance of AOB were indirectly driven by changes in ammonium availability. Ammonium is the main substrate for ammonia-oxidizing organisms (He et al., 2007; Nicol et al., 2008; Verhamme et al., 2011). Thus, compared with the other microsites, the positive effect of ant nests and grasses on ammonium contents observed here and in previous studies (Pêtal, 1998; Lenoir et al., 2001; Wu et al., 2015) may help to explain the positive effect of these microsites on the abundance of AOB. Similarly, the positive effect of trees on AOB may be indirectly mediated by the highest soil organic matter content found in this microsite (vs. other microsites evaluated), which, in turn, increases the availability of ammonium. Conversely, the negative impact of open areas on soil organic matter and ammonium availability may explain the low abundance of these microorganisms found in this microsite.

Interestingly, N-fixing shrubs and biocrusts were the only two microsites showing direct effects on AOB abundance in our SEMs (**Figures 4B,E**). One of these mechanisms could be synergistic effects between microbial communities involved in N cycling that thrive under N-fixing shrubs microsites (Zhang et al., 2015). For example, N-fixing shrubs are well-known to increase the abundance of microorganisms related to N fixing such as *Rhizobium* spp., which may promote the abundance and diversity of AOB by providing higher concentrations of ammonia to these microorganisms (Zhang et al., 2015). The observed indirect positive effect of N-fixing shrubs on nitrate availability via abundance of AOB (**Figure 4**) further suggests that all the N fixed under N-fixing shrubs (i.e., ammonium) may be transformed directly to nitrate, as suggested by the negative direct effect of this microsite on ammonium availability and by the typical high nitrification rates reported for N-fixing shrubs in drylands (e.g., Delgado-Baquerizo and Gallardo, 2011; Delgado-Baquerizo et al., 2011). Conversely, biocrust was the only microsite negatively affecting the abundance of AOB. Thus, while all the effects of open areas on AOB abundance are indirectly driven via the lowest soil C and ammonium availability found in this microsite, other unmeasured factors may indirectly drive the low AOB abundance under biocrusts. For instance, biocrusts often had a higher amount of soil phenols than open areas (Delgado-Baquerizo et al., 2015), which known allelopathic effects on particular soil bacterial communities (Herbert and Bertsch, 1995; Zsolnay, 1996; Saenz et al., 2006), and this could explain, at least in part, the direct negative effect of biocrusts on the abundance of AOB.

Indirect effects of aridity on soil properties such as pH may explain the highest abundance of AOA in the most arid locations. For example, we found a positive indirect effect of aridity on the abundance of AOA mediated by soil pH, which is supported by previous studies suggesting links between soil pH and the dominance of AOA (Nicol et al., 2008; Moin et al., 2009; Gubry-Rangin et al., 2011; Delgado-Baquerizo et al., 2013b). In addition, and contrary to what we found with AOB, the availability of ammonium had a negative direct effect on the abundance of AOA. Negative effect of ammonium availability on the abundance of AOA has been previously reported under laboratory conditions (from ∼155 ug N/g of soil; Verhamme et al., 2011). Both the large positive and direct effects of aridity and the negative direct effect of ammonium availability on the abundance of amoA genes from AOA support the notion that these microorganisms may outcompete AOB under oligotrophic conditions due to their high resistance to water and nutrient stress (Adair and Schwartz, 2008; Verhamme et al., 2011). This would allow AOA to carry out nitrification under the most unfavorable environmental conditions.

Taken together, our results indicate that soil microsite differentiation results in altered abundance of AOB along aridity gradients. The abundance of amoA genes from AOA is mainly driven by aridity in the drylands studied. These results are linked to indirect effects of microsite differentiation on amoA genes from AOB mediated by changes in soil properties such as soil carbon and ammonium availability, and to direct effects of aridity on amoA genes from AOA. These findings advance our understanding about how biotic and abiotic features control small-scale variations in microbial abundance and associated ecosystem processes in highly heterogeneous ecosystems such as drylands. They also indicate that identifying the main microsites promoting the abundance of particular microbial communities is particularly relevant for understanding how ongoing climate change may affect ecosystem functioning in a warmer and drier world.

## Author Contributions

MD-B designed this study in consultation with DJE, FTM, and BKS. Field data was collected by MD-B and DJE. Laboratory and statistical analyses were done by MD-B. The first draft of this paper was written by MD-B and all co-authors significantly contributed to improve it.

## Conflict of Interest Statement

The authors declare that the research was conducted in the absence of any commercial or financial relationships that could be construed as a potential conflict of interest.
