# Supplementary material for: Microsite Differentiation Drives the Abundance of Soil Ammonia Oxidizing Bacteria along Aridity Gradients
Source: Front Microbiol. 2016 Apr 18;7:505. doi: 10.3389/fmicb.2016.00505 (PMC4834304; doi:10.3389/fmicb.2016.00505)
Supplement: Supplementary file 1 [file Table_1.DOC]

**Table S1.** Main soil properties and AOA and AOB abundance for the different microsites included in this study in arid (n = 12) and mesic (n = 9) biomes.

| **Site** | **Microsite** | **Latitude^a^** | **Longitude^a^** | **Biome** | **AI^b^** | **pH** | **AOA^c^** | **AOB^c^** | **NH_4_^+d^** | **NO_3_^-d^** | **OC^e^** | **CN** |
| --- | --- | --- | --- | --- | --- | --- | --- | --- | --- | --- | --- | --- |
| **BU1** | **SHRUB** | -34.13 | 142.08 | Arid | 0.19 | 6.48 | 1.42E+09 | 1.08E+08 | 1.18 | 3.58 | 1.69 | 1.94 |
| **BU1** | **OPEN** | -34.13 | 142.08 | Arid | 0.19 | 7.63 | 5.25E+08 | 2.24E+07 | 0.00 | 1.11 | 0.75 | 1.87 |
| **BU1** | **BIOCRUST** | -34.13 | 142.08 | Arid | 0.19 | 6.06 | 4.94E+08 | 2.04E+07 | 0.20 | 3.74 | 0.79 | 2.19 |
| **BU1** | **TREE** | -34.13 | 142.08 | Arid | 0.19 | 7.33 | 9.15E+08 | 4.99E+07 | 0.90 | 0.00 | 1.08 | 2.48 |
| **BU1** | **GRASS** | -34.13 | 142.08 | Arid | 0.19 | 8.16 | 1.25E+09 | 6.73E+07 | 4.22 | 0.90 | 0.75 | 1.48 |
| **BU1** | **ANT NEST** | -34.13 | 142.08 | Arid | 0.19 | 8.06 | 1.34E+09 | 2.68E+08 | 4.47 | 8.24 | 1.45 | 1.53 |
| **BU2** | **SHRUB** | -34.16 | 142.20 | Arid | 0.20 | 9.13 | 7.75E+08 | 1.13E+08 | 0.12 | 10.09 | 0.80 | 2.36 |
| **BU2** | **OPEN** | -34.16 | 142.20 | Arid | 0.20 | 8.97 | 5.36E+08 | 3.17E+07 | 0.00 | 3.39 | 0.97 | 1.83 |
| **BU2** | **BIOCRUST** | -34.16 | 142.20 | Arid | 0.20 | 8.94 | 7.96E+08 | 3.01E+07 | 0.69 | 6.04 | 0.83 | 1.72 |
| **BU2** | **TREE** | -34.16 | 142.20 | Arid | 0.20 | 9.00 | 6.31E+08 | 5.44E+07 | 1.27 | 0.62 | 1.55 | 1.82 |
| **BU2** | **GRASS** | -34.16 | 142.20 | Arid | 0.20 | 8.35 | 4.66E+08 | 5.78E+07 | 12.07 | 4.89 | 1.24 | 2.27 |
| **BU2** | **ANT NEST** | -34.16 | 142.20 | Arid | 0.20 | 8.21 | 6.83E+08 | 3.91E+08 | 77.52 | 53.02 | 1.41 | 1.18 |
| **Site 1** | **SHRUB** | -34.00 | 145.73 | Arid | 0.29 | 5.85 | 6.82E+08 | 6.17E+07 | 1.35 | 2.09 | 1.43 | 1.27 |
| **Site 1** | **OPEN** | -34.00 | 145.73 | Arid | 0.29 | 6.28 | 7.80E+08 | 4.16E+07 | 0.00 | 1.11 | 0.89 | 1.66 |
| **Site 1** | **BIOCRUST** | -34.00 | 145.73 | Arid | 0.29 | 7.03 | 6.63E+08 | 4.47E+07 | 0.57 | 1.59 | 1.19 | 1.75 |
| **Site 1** | **TREE** | -34.00 | 145.73 | Arid | 0.29 | 6.36 | 1.45E+09 | 1.18E+08 | 2.99 | 3.05 | 3.03 | 1.09 |
| **Site 1** | **GRASS** | -34.00 | 145.73 | Arid | 0.29 | 8.80 | 4.38E+08 | 4.62E+07 | 2.66 | 0.91 | 2.23 | 1.34 |
| **Site 1** | **ANT NEST** | -34.00 | 145.73 | Arid | 0.29 | 9.07 | 3.62E+08 | 4.76E+07 | 3.81 | 16.08 | 1.19 | 0.77 |
| **Site 2** | **SHRUB** | -34.25 | 146.07 | Arid | 0.30 | 6.51 | 8.17E+08 | 3.05E+07 | 1.51 | 2.93 | 1.58 | 1.31 |
| **Site 2** | **OPEN** | -34.25 | 146.07 | Arid | 0.30 | 6.48 | 4.62E+08 | 1.37E+07 | 3.57 | 6.73 | 1.40 | 1.56 |
| **Site 2** | **BIOCRUST** | -34.25 | 146.07 | Arid | 0.30 | 6.10 | 6.48E+08 | 4.50E+07 | 0.81 | 6.42 | 1.43 | 1.25 |
| **Site 2** | **TREE** | -34.25 | 146.07 | Arid | 0.30 | 8.90 | 3.12E+08 | 2.24E+07 | 5.09 | 4.75 | 2.53 | 1.14 |
| **Site 2** | **GRASS** | -34.25 | 146.07 | Arid | 0.30 | 6.38 | 4.36E+08 | 4.14E+07 | 5.74 | 6.06 | 1.83 | 1.33 |
| **Site 2** | **ANT NEST** | -34.25 | 146.07 | Arid | 0.30 | 6.82 | 4.23E+08 | 6.25E+07 | 75.34 | 37.10 | 1.84 | 1.49 |
| **JM055** | **SHRUB** | -34.36 | 146.21 | Arid | 0.32 | 6.11 | 3.19E+08 | 1.05E+08 | 4.84 | 17.15 | 2.05 | 1.17 |
| **JM055** | **OPEN** | -34.36 | 146.21 | Arid | 0.32 | 6.91 | 1.40E+08 | 5.56E+07 | 3.11 | 7.64 | 2.21 | 1.30 |
| **JM055** | **BIOCRUST** | -34.36 | 146.21 | Arid | 0.32 | 6.68 | 4.60E+08 | 6.78E+07 | 3.85 | 3.93 | 1.98 | 1.08 |
| **JM055** | **TREE** | -34.36 | 146.21 | Arid | 0.32 | 8.47 | 8.02E+08 | 1.29E+08 | 5.66 | 5.82 | 4.16 | 0.94 |
| **JM055** | **GRASS** | -34.36 | 146.21 | Arid | 0.32 | 6.61 | 5.66E+08 | 1.27E+08 | 11.29 | 7.91 | 3.98 | 1.44 |
| **JM055** | **ANT NEST** | -34.36 | 146.21 | Arid | 0.32 | 6.52 | 2.19E+08 | 1.36E+08 | 8.46 | 11.80 | 2.42 | 1.21 |
| **JM057** | **SHRUB** | -34.42 | 146.31 | Arid | 0.32 | 6.61 | 4.92E+08 | 1.11E+08 | 5.21 | 13.68 | 2.44 | 1.02 |
| **JM057** | **OPEN** | -34.42 | 146.31 | Arid | 0.32 | 5.64 | 3.92E+08 | 3.73E+07 | 2.74 | 8.14 | 2.26 | 1.52 |
| **JM057** | **BIOCRUST** | -34.42 | 146.31 | Arid | 0.32 | 6.34 | 2.50E+07 | 2.37E+06 | 1.96 | 2.66 | 1.65 | 1.57 |
| **JM057** | **TREE** | -34.42 | 146.31 | Arid | 0.32 | 8.93 | 6.69E+08 | 1.73E+08 | 9.69 | 6.68 | 4.93 | 4.00 |
| **JM057** | **GRASS** | -34.42 | 146.31 | Arid | 0.32 | 5.94 | 8.89E+08 | 1.05E+08 | 7.51 | 4.34 | 2.74 | 1.19 |
| **JM057** | **ANT NEST** | -34.42 | 146.31 | Arid | 0.32 | 6.16 | 1.67E+08 | 6.73E+07 | 7.14 | 23.63 | 2.11 | 3.06 |
| **JM060** | **SHRUB** | -34.28 | 146.58 | Arid | 0.33 | 6.14 | 3.50E+08 | 6.42E+07 | 5.70 | 0.57 | 2.67 | 2.02 |
| **JM060** | **OPEN** | -34.28 | 146.58 | Arid | 0.33 | 5.85 | 6.46E+07 | 1.30E+07 | 1.02 | 6.30 | 0.84 | 0.57 |
| **JM060** | **BIOCRUST** | -34.28 | 146.58 | Arid | 0.33 | 5.97 | 3.14E+08 | 4.06E+07 | 1.92 | 6.27 | 2.58 | 0.81 |
| **JM060** | **TREE** | -34.28 | 146.58 | Arid | 0.33 | 6.44 | 1.18E+09 | 2.10E+08 | 4.55 | 2.04 | 2.42 | 2.28 |
| **JM060** | **GRASS** | -34.28 | 146.58 | Arid | 0.33 | 6.04 | 4.65E+08 | 6.23E+07 | 4.43 | 3.49 | 1.40 | 1.01 |
| **JM060** | **ANT NEST** | -34.28 | 146.58 | Arid | 0.33 | 7.61 | 3.67E+08 | 5.44E+07 | 14.82 | 7.08 | 1.72 | 1.31 |
| **JM061** | **SHRUB** | -34.35 | 146.92 | Arid | 0.39 | 5.84 | 7.45E+08 | 1.03E+08 | 4.10 | 4.96 | 8.90 | 5.84 |
| **JM061** | **OPEN** | -34.35 | 146.92 | Arid | 0.39 | 5.50 | 1.77E+07 | 1.42E+07 | 3.36 | 0.00 | 1.82 | 0.98 |
| **JM061** | **BIOCRUST** | -34.35 | 146.92 | Arid | 0.39 | 5.74 | 5.89E+08 | 7.60E+07 | 6.07 | 3.40 | 1.95 | 1.04 |
| **JM061** | **TREE** | -34.35 | 146.92 | Arid | 0.39 | 8.66 | 3.74E+08 | 4.38E+07 | 9.61 | 4.21 | 1.74 | 0.27 |
| **JM061** | **GRASS** | -34.35 | 146.92 | Arid | 0.39 | 6.45 | 1.00E+08 | 1.04E+07 | 5.05 | 5.02 | 6.03 | 3.46 |
| **JM061** | **ANT NEST** | -34.35 | 146.92 | Arid | 0.39 | 6.19 | 2.67E+08 | 6.26E+07 | 6.48 | 3.99 | 1.87 | 0.83 |
| **JM062** | **SHRUB** | -34.44 | 147.42 | Arid | 0.44 | 5.62 | 4.94E+07 | 4.61E+07 | 0.69 | 3.11 | 2.92 | 1.77 |
| **JM062** | **OPEN** | -34.44 | 147.42 | Arid | 0.44 | 5.51 | 7.84E+07 | 2.22E+07 | 5.74 | 6.10 | 4.49 | 2.28 |
| **JM062** | **BIOCRUST** | -34.44 | 147.42 | Arid | 0.44 | 5.68 | 3.72E+07 | 5.03E+07 | 2.62 | 1.46 | 3.80 | 2.34 |
| **JM062** | **TREE** | -34.44 | 147.42 | Arid | 0.44 | 7.07 | 7.14E+07 | 8.27E+07 | 45.55 | 8.70 | 9.66 | 3.98 |
| **JM062** | **GRASS** | -34.44 | 147.42 | Arid | 0.44 | 5.38 | 1.29E+08 | 1.12E+08 | 8.33 | 1.59 | 4.96 | 1.40 |
| **JM062** | **ANT NEST** | -34.44 | 147.42 | Arid | 0.44 | 5.37 | 8.11E+07 | 7.44E+07 | 10.14 | 3.31 | 4.43 | 3.16 |
| **JM092** | **SHRUB** | -33.32 | 148.16 | Arid | 0.46 | 6.47 | 6.35E+08 | 3.84E+07 | 3.81 | 3.65 | 1.38 | 1.70 |
| **JM092** | **OPEN** | -33.32 | 148.16 | Arid | 0.46 | 5.83 | 2.90E+08 | 3.49E+07 | 1.84 | 8.36 | 2.07 | 1.55 |
| **JM092** | **BIOCRUST** | -33.32 | 148.16 | Arid | 0.46 | 6.55 | 5.58E+08 | 4.38E+07 | 4.10 | 2.99 | 1.84 | 1.70 |
| **JM092** | **TREE** | -33.32 | 148.16 | Arid | 0.46 | 8.48 | 3.63E+08 | 1.13E+08 | 16.75 | 16.94 | 8.82 | 1.86 |
| **JM092** | **GRASS** | -33.32 | 148.16 | Arid | 0.46 | 7.27 | 9.48E+08 | 8.18E+07 | 7.43 | 8.58 | 6.91 | 1.64 |
| **JM092** | **ANT NEST** | -33.32 | 148.16 | Arid | 0.46 | 6.07 | 1.17E+08 | 2.91E+07 | 4.68 | 15.90 | 2.10 | 2.21 |
| **JM081** | **SHRUB** | -33.51 | 148.17 | Arid | 0.47 | 5.83 | 2.25E+08 | 1.06E+08 | 25.09 | 21.40 | 6.50 | 1.53 |
| **JM081** | **OPEN** | -33.51 | 148.17 | Arid | 0.47 | 5.70 | 1.16E+08 | 2.47E+07 | 11.29 | 11.11 | 3.00 | 1.35 |
| **JM081** | **BIOCRUST** | -33.51 | 148.17 | Arid | 0.47 | 5.74 | 7.25E+08 | 6.71E+07 | 3.48 | 4.61 | 2.89 | 1.43 |
| **JM081** | **TREE** | -33.51 | 148.17 | Arid | 0.47 | 8.10 | 2.89E+07 | 2.99E+06 | 19.30 | 5.20 | 9.08 | 2.00 |
| **JM081** | **GRASS** | -33.51 | 148.17 | Arid | 0.47 | 5.64 | 4.49E+08 | 7.16E+07 | 5.78 | 6.43 | 3.03 | 1.36 |
| **JM081** | **ANT NEST** | -33.51 | 148.17 | Arid | 0.47 | 6.25 | 5.94E+08 | 7.59E+07 | 6.61 | 4.28 | 2.41 | 1.74 |
| **JM085** | **SHRUB** | -33.73 | 148.20 | Arid | 0.49 | 5.90 | 4.93E+08 | 6.80E+07 | 2.83 | 9.98 | 3.22 | 1.99 |
| **JM085** | **OPEN** | -33.73 | 148.20 | Arid | 0.49 | 5.91 | 1.98E+08 | 5.15E+07 | 1.92 | 0.00 | 1.39 | 1.57 |
| **JM085** | **BIOCRUST** | -33.73 | 148.20 | Arid | 0.49 | 6.54 | 1.54E+08 | 4.51E+07 | 0.81 | 3.13 | 1.67 | 1.53 |
| **JM085** | **TREE** | -33.73 | 148.20 | Arid | 0.49 | 7.35 | 7.61E+08 | 6.61E+07 | 6.40 | 17.47 | 4.74 | 1.43 |
| **JM085** | **GRASS** | -33.73 | 148.20 | Arid | 0.49 | 5.85 | 2.90E+08 | 5.33E+07 | 4.92 | 6.79 | 2.56 | 2.13 |
| **JM085** | **ANT NEST** | -33.73 | 148.20 | Arid | 0.49 | 6.41 | 4.85E+08 | 8.58E+07 | 4.68 | 39.07 | 3.04 | 1.81 |
| **JM079** | **SHRUB** | -33.83 | 148.61 | Mesic | 0.51 | 6.44 | 3.59E+08 | 9.44E+07 | 4.96 | 3.18 | 3.03 | 1.79 |
| **JM079** | **OPEN** | -33.83 | 148.61 | Mesic | 0.51 | 5.86 | 2.24E+08 | 3.02E+07 | 4.43 | 2.66 | 1.73 | 1.33 |
| **JM079** | **BIOCRUST** | -33.83 | 148.61 | Mesic | 0.51 | 5.58 | 6.42E+08 | 5.12E+07 | 1.96 | 2.07 | 2.20 | 1.49 |
| **JM079** | **TREE** | -33.83 | 148.61 | Mesic | 0.51 | 7.24 | 8.34E+08 | 1.44E+08 | 16.55 | 11.34 | 7.32 | 1.74 |
| **JM079** | **GRASS** | -33.83 | 148.61 | Mesic | 0.51 | 5.64 | 7.95E+07 | 5.81E+07 | 11.58 | 1.78 | 2.06 | 1.32 |
| **JM079** | **ANT NEST** | -33.83 | 148.61 | Mesic | 0.51 | 5.77 | 1.03E+08 | 1.93E+07 | 10.59 | 8.43 | 1.68 | 1.48 |
| **JM065** | **SHRUB** | -34.74 | 149.89 | Mesic | 0.57 | 5.91 | 4.49E+07 | 6.90E+07 | 5.66 | 1.34 | 2.87 | 1.41 |
| **JM065** | **OPEN** | -34.74 | 149.89 | Mesic | 0.57 | 5.43 | 5.20E+07 | 9.65E+07 | 8.21 | 9.49 | 2.23 | 2.27 |
| **JM065** | **BIOCRUST** | -34.74 | 149.89 | Mesic | 0.57 | 5.35 | 2.34E+07 | 4.74E+07 | 3.40 | 6.75 | 2.08 | 1.79 |
| **JM065** | **TREE** | -34.74 | 149.89 | Mesic | 0.57 | 5.52 | 2.71E+07 | 7.04E+07 | 10.84 | 4.02 | 4.87 | 1.92 |
| **JM065** | **GRASS** | -34.74 | 149.89 | Mesic | 0.57 | 5.54 | 1.33E+07 | 2.77E+07 | 17.90 | 1.35 | 4.30 | 2.08 |
| **JM065** | **ANT NEST** | -34.74 | 149.89 | Mesic | 0.57 | 6.13 | 2.11E+07 | 1.80E+07 | 4.88 | 6.65 | 1.64 | 2.17 |
| **JM076** | **SHRUB** | -34.36 | 148.92 | Mesic | 0.57 | 5.49 | 4.05E+07 | 9.32E+07 | 9.36 | 12.54 | 5.48 | 1.57 |
| **JM076** | **OPEN** | -34.36 | 148.92 | Mesic | 0.57 | 5.61 | 4.67E+07 | 1.23E+08 | 13.10 | 1.67 | 2.58 | 1.47 |
| **JM076** | **BIOCRUST** | -34.36 | 148.92 | Mesic | 0.57 | 6.63 | 2.38E+07 | 5.23E+07 | 9.44 | 2.91 | 1.72 | 1.76 |
| **JM076** | **TREE** | -34.36 | 148.92 | Mesic | 0.57 | 5.12 | 5.60E+07 | 1.34E+08 | 10.63 | 1.99 | 3.87 | 2.10 |
| **JM076** | **GRASS** | -34.36 | 148.92 | Mesic | 0.57 | 5.73 | 2.74E+07 | 7.54E+07 | 26.74 | 3.02 | 6.17 | 1.96 |
| **JM076** | **ANT NEST** | -34.36 | 148.92 | Mesic | 0.57 | 5.60 | 3.27E+07 | 6.49E+07 | 7.10 | 6.90 | 2.58 | 1.62 |
| **JM077** | **SHRUB** | -33.98 | 148.95 | Mesic | 0.62 | 7.30 | 1.40E+08 | 1.46E+07 | 5.50 | 10.92 | 6.59 | 1.89 |
| **JM077** | **OPEN** | -33.98 | 148.95 | Mesic | 0.62 | 6.43 | 2.32E+08 | 7.43E+07 | 0.90 | 5.05 | 0.90 | 1.27 |
| **JM077** | **BIOCRUST** | -33.98 | 148.95 | Mesic | 0.62 | 6.06 | 9.71E+07 | 5.26E+07 | 2.83 | 6.46 | 1.34 | 1.68 |
| **JM077** | **TREE** | -33.98 | 148.95 | Mesic | 0.62 | 6.18 | 8.49E+07 | 9.63E+07 | 1.64 | 7.42 | 2.45 | 1.83 |
| **JM077** | **GRASS** | -33.98 | 148.95 | Mesic | 0.62 | 6.50 | 1.30E+08 | 2.41E+07 | 3.36 | 5.33 | 1.66 | 1.30 |
| **JM077** | **ANT NEST** | -33.98 | 148.95 | Mesic | 0.62 | 6.56 | 3.39E+08 | 6.95E+07 | 21.11 | 21.54 | 1.52 | 1.18 |
| **Site 3** | **SHRUB** | -33.91 | 150.99 | Mesic | 0.66 | 6.05 | 1.31E+08 | 3.91E+07 | 20.25 | 13.58 | 5.42 | 1.20 |
| **Site 3** | **OPEN** | -33.91 | 150.99 | Mesic | 0.66 | 5.97 | 2.20E+08 | 2.99E+07 | 7.84 | 19.46 | 7.69 | 1.59 |
| **Site 3** | **BIOCRUST** | -33.91 | 150.99 | Mesic | 0.66 | 7.04 | 1.55E+08 | 5.29E+07 | 2.79 | 14.00 | 4.41 | 1.75 |
| **Site 3** | **TREE** | -33.91 | 150.99 | Mesic | 0.66 | 6.00 | 4.32E+08 | 4.01E+07 | 11.45 | 18.95 | 5.47 | 1.14 |
| **Site 3** | **GRASS** | -33.91 | 150.99 | Mesic | 0.66 | 5.43 | 1.50E+08 | 4.58E+07 | 14.33 | 23.20 | 5.56 | 1.32 |
| **Site 3** | **ANT NEST** | -33.91 | 150.99 | Mesic | 0.66 | 5.69 | 1.03E+08 | 6.86E+07 | 1.14 | 70.03 | 5.08 | 1.24 |
| **JM100** | **SHRUB** | -34.51 | 150.24 | Mesic | 0.71 | 5.63 | 2.20E+07 | 4.48E+07 | 5.25 | 6.69 | 2.65 | 2.71 |
| **JM100** | **OPEN** | -34.51 | 150.24 | Mesic | 0.71 | 6.64 | 4.57E+07 | 4.79E+07 | 2.62 | 4.93 | 2.35 | 4.13 |
| **JM100** | **BIOCRUST** | -34.51 | 150.24 | Mesic | 0.71 | 6.36 | 1.86E+07 | 2.47E+07 | 0.65 | 0.50 | 1.71 | 3.50 |
| **JM100** | **TREE** | -34.51 | 150.24 | Mesic | 0.71 | 5.93 | 2.76E+07 | 3.67E+07 | 1.68 | 3.36 | 2.88 | 3.18 |
| **JM100** | **GRASS** | -34.51 | 150.24 | Mesic | 0.71 | 5.55 | 3.25E+07 | 4.28E+07 | 5.50 | 3.20 | 2.77 | 3.00 |
| **JM100** | **ANT NEST** | -34.51 | 150.24 | Mesic | 0.71 | 5.74 | 3.48E+07 | 4.48E+07 | 2.58 | 1.36 | 1.92 | 2.80 |
| **JM072** | **SHRUB** | -33.62 | 150.77 | Mesic | 0.71 | 5.77 | 6.33E+07 | 7.49E+07 | 1.59 | 29.45 | 2.84 | 1.16 |
| **JM072** | **OPEN** | -33.62 | 150.77 | Mesic | 0.71 | 5.55 | 7.59E+07 | 5.86E+07 | 8.87 | 4.85 | 2.55 | 1.68 |
| **JM072** | **BIOCRUST** | -33.62 | 150.77 | Mesic | 0.71 | 5.50 | 1.32E+08 | 5.99E+07 | 6.57 | 9.30 | 2.37 | 1.59 |
| **JM072** | **TREE** | -33.62 | 150.77 | Mesic | 0.71 | 5.63 | 4.23E+07 | 1.59E+08 | 13.43 | 8.38 | 8.45 | 3.14 |
| **JM072** | **GRASS** | -33.62 | 150.77 | Mesic | 0.71 | 5.61 | 4.74E+07 | 6.83E+07 | 3.16 | 13.99 | 1.85 | 1.45 |
| **JM072** | **ANT NEST** | -33.62 | 150.77 | Mesic | 0.71 | 6.06 | 9.12E+07 | 1.10E+08 | 12.07 | 2.93 | 5.70 | 2.34 |
| **JM071** | **SHRUB** | -33.65 | 150.86 | Mesic | 0.76 | 5.47 | 8.27E+07 | 6.33E+07 | 10.84 | 7.18 | 2.11 | 1.43 |
| **JM071** | **OPEN** | -33.65 | 150.86 | Mesic | 0.76 | 5.23 | 1.92E+08 | 5.82E+07 | 2.17 | 0.00 | 1.96 | 1.41 |
| **JM071** | **BIOCRUST** | -33.65 | 150.86 | Mesic | 0.76 | 5.27 | 2.90E+07 | 1.31E+07 | 7.06 | 3.19 | 2.28 | 2.22 |
| **JM071** | **TREE** | -33.65 | 150.86 | Mesic | 0.76 | 5.46 | 4.88E+08 | 1.68E+08 | 13.02 | 14.96 | 3.46 | 1.44 |
| **JM071** | **GRASS** | -33.65 | 150.86 | Mesic | 0.76 | 5.93 | 1.11E+08 | 4.34E+07 | 13.30 | 2.25 | 1.99 | 0.88 |
| **JM071** | **ANT NEST** | -33.65 | 150.86 | Mesic | 0.76 | 6.12 | 3.51E+07 | 4.13E+07 | 35.12 | 8.63 | 2.12 | 1.56 |
| **Site 4** | **SHRUB** | -33.98 | 151.06 | Mesic | 0.80 | 5.78 | 2.54E+07 | 2.75E+07 | 6.07 | 1.25 | 3.10 | 3.08 |
| **Site 4** | **OPEN** | -33.98 | 151.06 | Mesic | 0.80 | 5.12 | 6.88E+07 | 4.89E+07 | 3.57 | 2.66 | 2.92 | 2.11 |
| **Site 4** | **BIOCRUST** | -33.98 | 151.06 | Mesic | 0.80 | 7.10 | 4.34E+06 | 4.47E+06 | 3.57 | 0.60 | 1.18 | 5.04 |
| **Site 4** | **TREE** | -33.98 | 151.06 | Mesic | 0.80 | 4.83 | 4.46E+07 | 5.18E+07 | 27.44 | 0.73 | 12.32 | 2.00 |
| **Site 4** | **GRASS** | -33.98 | 151.06 | Mesic | 0.80 | 5.05 | 6.27E+06 | 5.52E+06 | 6.73 | 2.01 | 2.34 | 2.46 |
| **Site 4** | **ANT NEST** | -33.98 | 151.06 | Mesic | 0.80 | 5.33 | 1.41E+07 | 1.61E+07 | 7.30 | 2.07 | 1.64 | 2.33 |

^a^Degrees

^b^Precipitation/potential evapotranspiration

^c^Number of copies g^-1^ soil

^d^mg N kg^-1^ soil

^e^%
